# Marginal Fit of Three Commonly Used Veneers for Smile Enhancement: An *In Vitro* Study

**DOI:** 10.1055/s-0045-1810443

**Published:** 2025-08-05

**Authors:** Vinod Bandela, Shital Sonune, Ram Basany, Bharathi Munagapati, Saif Faruqi, Saraswathi Kanaparthi, Reef Basher Saad Alkayid, Wroud Alturqi Alshammari, Alreem Abdulaziz Alnuman, Eatedal Mukhlef Alruwaili, Miad Abdulnasser Alahmed, Almas Bassam Aljoufi, Haya Abdulrahman Alrayes, Munirah Saleh Alabid, Doaa Abdelaziz A. Helal

**Affiliations:** 1 Department of Prosthetic Dental Sciences, College of Dentistry, Jouf University, Al Jouf, Saudi Arabia; 2 Department of Prosthodontics, SVS Institute of Dental Sciences, Mahbubnagar, Telangana, India; 3 Department of Prosthodontics, G PullaReddy Dental College & Hospital, Kurnool, Andhra Pradesh, India; 4 McCabe Dentures and Implant Solutions, Cambridge, Ontario, Canada; 5 Pedodontist, Private Practitioner, Hyderabad, Telangana, India; 6 Fixed Prosthodontics Department, College of Dentistry, Jouf University, Al Jawf Region, Saudi Arabia; 7 Fixed Prosthodontics Department, College of Dentistry, Beni Suef University, Egypt

**Keywords:** esthetics, indirect composites, pressable ceramics, CAD/CAM, marginal fit

## Abstract

**Objectives:**

The objective of the present study was to evaluate the marginal fit of three most commonly used veneers in dentistry.

**Materials and Methods:**

A maxillary central incisor was embedded in a self-cure acrylic resin block, with the crown and 2 mm of the root exposed to facilitate standardized tooth preparation. Following the preparation, 30 elastomeric impressions were made to produce master casts. These working dies were then randomly allocated to three experimental groups, each consisting of 15 samples. Group I comprised indirect composite veneers (ICV), fabricated using laboratory composite resin. Group II included pressable ceramic veneers (PCV), manufactured using heat-pressed lithium disilicate. Group III consisted of computer-aided designing (CAD)/computer-aided manufacturing (CAM) ceramic veneers, fabricated through digital milling of lithium disilicate blocks. All veneers were fabricated in accordance with the respective manufacturer's guidelines and were carefully repositioned on the prepared tooth to evaluate the marginal discrepancy using a stereomicroscope.

**Statistical Analysis:**

Three pre-designated points—mesio-labial, mid-labial, and disto-labial and mesio-palatal, mid-palatal, and disto-palatal on the labial and palatal margins—were measured. The values were recorded and analyzed with one-way ANOVA and Tukey's post-hoc test using SPSS software.

**Results:**

ICV showed more variation with mean discrepancy of 189.24 ± 25.17 µm at cervical margin and 79.01 ± 11.68 µm at palatal area. PCV showed less variation with mean discrepancy of 48.2 ± 8.35 µm and 40.58 ± 9.47 µm at cervical and palatal areas, respectively. CAD/CAM-fabricated ceramic veneers showed mean discrepancy of 94.24 ± 9.00 µm at cervical and 52.72 ± 16.33 µm at palatal areas.

**Conclusions:**

Pressable ceramic veneers showed the best marginal fit at both cervical and palatal margins followed by CAD/CAM veneers. Indirect composite veneers showed poorest marginal fit. The marginal discrepancy values were within the clinically acceptable range for PCV and CAD/CAM ceramic veneers.

**Clinical Significance:**

It is of paramount importance that the dentist should choose wisely the veneer material taking the marginal fit into account.

## Introduction

Imperfections in the appearance of anterior teeth can significantly affect a patient's self-confidence and perceived aesthetics. 1 The increasing demand for smile enhancement has led to a growing emphasis on minimally invasive cosmetic dental treatments, particularly for anterior teeth rehabilitation. Several treatment modalities such as enamel recontouring, bleaching, veneers, and full crowns have been utilized to improve aesthetic outcomes. 2 3 Historically, full-coverage crowns were considered the most durable solution; however, this approach is invasive, often requiring substantial removal of healthy tooth structure and potentially compromising pulp vitality and periodontal integrity. 4

In contrast, laminate veneers offer a more conservative approach for managing aesthetic concerns related to shape, color, alignment, and minor fractures of anterior teeth. Their application has broadened significantly due to advancements in adhesive technologies and restorative materials. Veneers are now commonly indicated for discoloration, enamel hypoplasia, diastemas, post-orthodontic corrections, and minor malpositioning. 5 6

The long-term clinical success of any indirect aesthetic restoration is determined by three critical parameters: aesthetics, mechanical strength, and marginal adaptation. Among these, marginal fit is of paramount importance as it directly influences the longevity of the restoration and the health of surrounding periodontal tissues. 7 Marginal fit refers to the vertical discrepancy between the margin of the veneer and the finish line of the prepared tooth. Inadequate marginal adaptation can lead to cement dissolution, microleakage, secondary caries, and periodontal inflammation. 8

Several authors have attempted to define the clinically acceptable range of marginal discrepancies. Recent literature suggests that marginal gaps ≤120 µm are acceptable, although ideal ranges may vary depending on the material and clinical context. 9 10 A range of 50 to 120 µm has been frequently cited as optimal, with gaps exceeding this threshold associated with increased clinical complications. 11

Marginal fit is influenced by multiple factors including preparation geometry, material properties, impression techniques, processing methods, and cementation protocols. Notably, advances in CAD/CAM systems have introduced new fabrication workflows that may impact marginal adaptation when compared with traditional heat-pressed ceramics or indirect composite systems. 12 13

Given the wide variety of veneer materials available, it becomes essential to evaluate and compare their marginal fit characteristics. The present *in vitro* study was designed to assess the vertical marginal discrepancy of three commonly used veneer types—indirect composite veneers (ICV), pressable ceramic veneers (PCV), and computer-aided designing (CAD)/computer-aided manufacturing (CAM) milled ceramic veneers (CAD)—at six designated points using stereomicroscopy. The objective was to determine which material provides the best marginal adaptation and whether differences fall within clinically acceptable thresholds.

## Materials and Methods

**Fig. 1:**
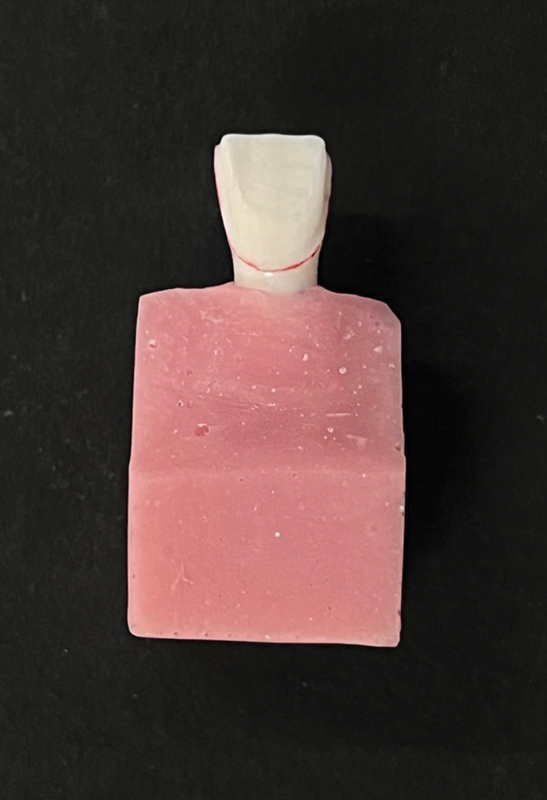
Master die embedded in acrylic.

In the current study, human maxillary central incisor was collected that was extracted due to periodontal reason. The selected tooth was cleaned, disinfected, and handled as per the recommendations and guidelines of the Occupational Safety and Health Administration (OSHA) and Centers for Disease Control and Prevention (CDC). The tooth was examined under stereomicroscope (Wuzhou New Found Instrument Co. Ltd, China; Model: XTL, 3400E, Magnification:15X) to check for cracks and/or surface defects. The tooth was later embedded in a self-cure acrylic resin block so that the crown and 2 mm portion of the root was exposed ( Fig. 1 ). A preoperative silicon index was made using polyvinyl siloxane impression material (Reprosil, Dentsply Caulk, USA) to gauge the reduction of the tooth after preparation.

The tooth was manually prepared using a depth cutting bur no. 4141 and a round end tapered fissure diamond bur no. 4138 (Microdont bur system, Sao Paulo, Brazil). The facial reduction was aimed at 0.7 mm, while simultaneously maintaining the contour of the tooth. A supragingival margin was prepared with a moderate chamfer as a finish line. The incisal edge was slightly reduced, and an incisal overlap design was preferred. The palatal margin was placed 2 mm apical to the incisal edge above the concavity. The finishing of tooth preparation was done using no. 2135 bur (Microdont bur system, Sao Paulo, Brazil). The prepared tooth was approximated in the preoperative silicon index to confirm and standardize the samples. A digital vernier caliper (Mitutoyo, RS Components & Controls (I) Ltd. Noida, India) was used to measure this distance followed by shade selection where A1 shade (Vitapan Classical shade guide, VITA Zahnfabrik, H. Rauter GmbH & Co. KG, Germany) was selected for all the selected samples.

Sample size ( *n* = 15 per group) was chosen based on prior *in vitro* studies evaluating marginal fit, to ensure sufficient power (80%) at α = 0.05 for detecting intergroup differences. The dies were randomly allocated to the three groups using a computer-generated random number sequence.

An incisal overlap design was selected for this study due to its favorable biomechanical and aesthetic characteristics. This design provides greater surface area for bonding, improves load distribution during function, and allows better control over incisal translucency and optical layering during fabrication. Although some studies suggest that butt–joint preparations may offer improved marginal fit due to simpler geometry and ease of adaptation, 14 the overlap design was intentionally chosen in this study to replicate the commonly preferred clinical protocol for aesthetically demanding anterior veneers and to enhance fracture resistance under functional loading. 15 16

For precise impression, a customized stainless-steel tray was fabricated to cover the acrylic resin block of the prepared tooth. In all 30 elastomeric impressions (Reprosil, Dentsply Caulk, USA) were made and duplicated into 30 working dies using Type IV dental stone (Ultrarock, Kalabhai Dental Pvt Ltd, Maharashtra, India). The working dies were randomly divided into three groups:

Group I: Indirect composite veneers (ICV) ( *n* = 15)
Group II: Pressable ceramic veneers (PCV) ( *n* = 15)
Group III: CAD/CAM milled ceramic veneers ( *n* = 15), where digital impressions were recorded from the prepared tooth with the help of system-specific handheld device (Primescan ^TM^ , Dentsply Sirona, USA) and milling of veneers was done using dental milling machine (Sirona Cerec MC XL, Dentsply Sirona, USA).

In Group I, ICV (SR Adoro, Ivoclar Vivadent, Schaan, Liechtenstein) were fabricated on their respective dies according to the manufacturer's instructions. A total of 15 ICV were acquired by this method.

In Group II, PCV were prepared using pressable ceramic furnace (Programat EP 5010 G2, Ivoclar Vivadent, Schaan, Liechtenstein). A wax pattern was made by conventional method on each stone die and wax patterns were invested. Preheated ceramic ingots (IPS e.max Press A1 shade; Ivoclar Vivadent, Schaan, Liechtenstein) were placed inside the ring and transferred to the furnace with pressure (0.3–0.4 MPa) to complete the pressing cycle. Once the ceramic substructure was obtained it was later stained and glazed using conventional layering technique. Later, they were divested in sandblasting unit (Sandblaster SR 922, Sirio Dental S.R.L, Meldola (FC) Italy) by using 50-μm glass particles followed by removal of the sprue. Likewise, a total of 15 samples were fabricated by similar technique.

All veneers were gently placed over the prepared tooth using non-traumatic tweezers under magnification to avoid any distortion or wear of the margin. Between repeated placements, the tooth was visually inspected to ensure that the preparation boundaries remained unchanged.

For the CAD/CAM group, a virtual cement spacer of 80 µm was set during digital design in accordance with manufacturer's guidelines (Dentsply Sirona, USA). This value aligns with the standard die spacer thickness used in laboratory protocols for indirect composite and ceramic veneers. Careful attention was paid to standardize this parameter across all groups to ensure comparability.

In Group III, multichromatic blocks (Multishade A1; Ivoclar Vivadent, Schaan, Liechtenstein) were used to mill 15 veneers (Sirona Cerec MC XL, Dentsply Sirona, USA). A 3D camera (Primescan ^TM^ , Dentsply Sirona, USA) was used to scan all the surfaces of the prepared tooth. The image was transferred into inLab CAD 18.0 software (Dentsply Sirona, USA) and preparation finish line was marked on the digital model. Contours were adjusted by labeling the curvature lines after selecting the required anatomy. Later, the program was directed toward milling of the block (e.max CAD Ivoclar Vivadent, Liechtenstein). The block was done using milling machine (Cerec MC XL, Dentsply Sirona, USA). The veneers thus obtained with an attached sprue was separated, covered with a glazing spray (e.max CAD Glaze spray, Ivoclar Vivadent, Liechtenstein) followed by sintering in the same furnace for 26 minutes. Using this technique 15 samples were obtained.

For the CAD/CAM group, a virtual cement spacer of 80 µm was set during digital design in accordance with manufacturer's guidelines (Dentsply Sirona, USA). This value aligns with the standard die spacer thickness used in laboratory protocols for indirect composite and ceramic veneers. Careful attention was paid to standardize this parameter across all groups to ensure comparability.

A virtual cement spacer of 80 µm was defined during the digital design phase in accordance with manufacturer's specifications (Dentsply Sirona, USA). This thickness is comparable to the standard die spacer settings used in conventional laboratory protocols for both composite and pressable ceramic veneers, ensuring uniformity across all groups with respect to internal relief for cementation.

All veneers were gently placed over the prepared tooth using non-traumatic tweezers under magnification to avoid any distortion or wear of the margin. Between repeated placements, the tooth was visually inspected to ensure that the preparation boundaries remained unchanged.

**Fig. 2:**
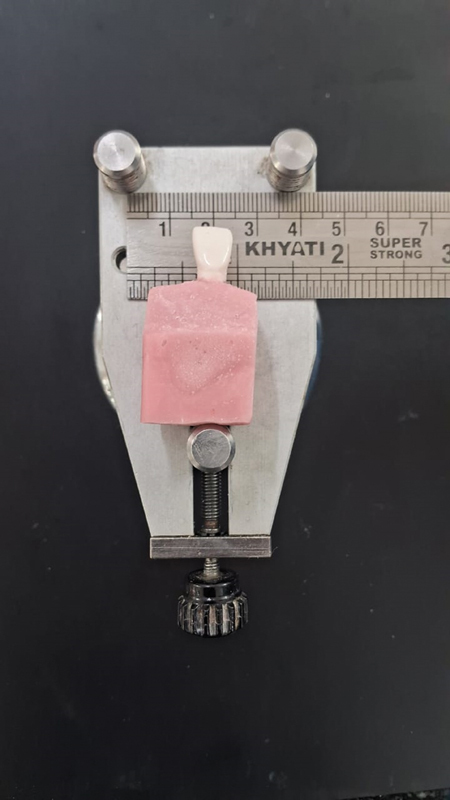
Positioning jig used during marginal fit evaluation.

**Fig. 3:**
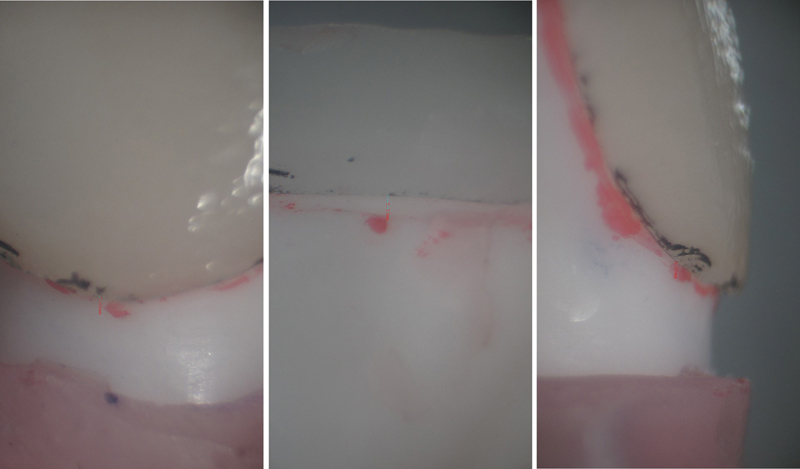
Discrepancy at the labial cervical margins of three study groups.

**Fig. 4:**
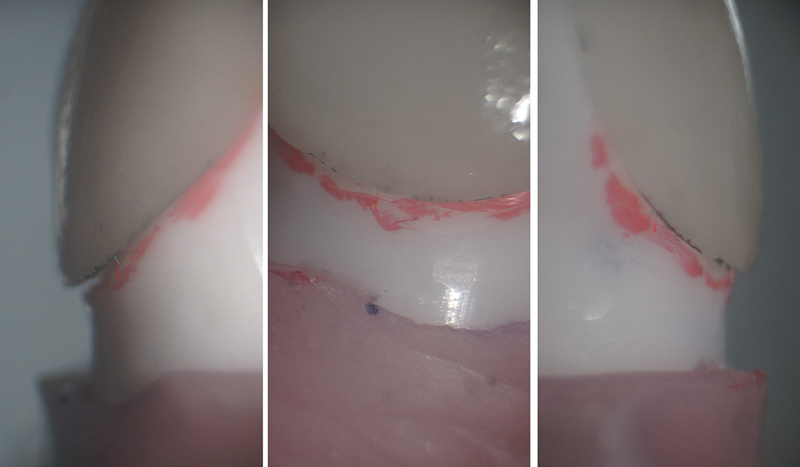
Discrepancy at palatal cervical margins in three study groups.

Veneers in each group were placed over the prepared tooth mounted in resin block that was stabilized on spring-loaded jig assembly ( Fig. 2 ). The marginal discrepancy on each sample was recorded under zoom stereomicroscope (XTL-3400E, Wuzhou New Found Instrument Co. Ltd, China) with 15× magnification. Three pre-designated points, mesio-labial (ML), mid-labial (MidL), and disto-labial (DL), were measured on the labial cervical margin of all the three groups ( Fig. 3 ). Likewise, three points at the palatal margins in all the groups at mesio-palatal (MP), mid-palatal (MidP), and disto-palatal (DP) were measured ( Fig. 4 ). The values of vertical marginal fit were calculated using Image Analysis System (IA44, PAXcam ^TM^ Camera, LECO, St. Joseph, MI, USA).

To prevent any distortion or alteration of the prepared tooth margins, especially at the gingival and cervical finish lines, all veneer placements and removals were performed using non-traumatic tweezers under 15× magnification. No mechanical adjustments or re-preparations were performed between samples. The prepared tooth was carefully inspected after each placement to ensure that the preparation boundaries remained intact throughout the study.

Prior to measurement, the stereomicroscope system was calibrated according to the manufacturer's specifications to ensure accuracy. All measurements were performed by a single trained operator using the same calibrated image analysis software, which minimized variability. As only one operator was involved, inter-examiner reliability was not assessed in this study.

A single experienced operator performed all tooth preparations, veneer placements, and marginal assessments to eliminate inter-operator variability. Image analysis was conducted by a blinded examiner using calibrated software to minimize measurement bias.

The mean values for each group were recorded and analyzed with one-way ANOVA and Tukey's post-hoc test using SPSS version 22.0 software.

## Results

The mean marginal discrepancy was found to be highest for ICV followed by CAD/CAM fabricated group and least for PCV group. ICV showed more variation with mean discrepancy of 189.24 ± 25.17 µm at cervical region and 79.01 ± 11.68 µm at palatal area. PCV showed less variation with mean discrepancy of 48.2 ± 8.35 µm and 40.58 ± 9.47 µm at cervical and palatal areas, respectively. CAD/CAM-fabricated ceramic veneers showed mean discrepancy of 94.24 ± 9.00 µm at cervical and 52.72 ± 16.33 µm at palatal areas ( Table 1 ). One-way ANOVA showed highly significant difference across the mean ( *p* < 0.0001) (Tables 1 and 2). At cervical and palatal margins, all the samples showed highly significant difference values ( *p* < 0.0001) ( Table 2 ). The difference between Group II and Group III was statistically significant ( *p* = 0.034), although smaller compared with Group I ( Table 2 ).

**Table 1:** Descriptive statistics of discrepancy at labial and palatal cervical margins

Groups	Discrepancy at labial cervical margin (Mean ± SD) µm	Discrepancy at palatal cervical margin (Mean ± SD) µm
Indirect composite veneers (ICV) (Group I)	189.24 ± 25.17	79.01 ± 11.68
Pressable ceramic veneers (PCV) (Group II)	48.2 ± 8.35	40.58 ± 9.47
CAD/CAM ceramic veneers (Group III)	94.24 ± 9.00	52.72 ± 16.33
*p* -Value	**<0.0001**	**<0.0001**

Abbreviations: SD, standard deviation; µm, micrometer.

Note: Bold *p* -Values indicate statistical significance.

**Table 2:** Pair-wise comparison of labial and palatal cervical margins discrepancy

Groups	Group I vs. Group II	Group I vs. Group III	Group II vs. Group III
*p* -Value for labial cervical margin	**<0.0001**	**<0.0001**	**<0.0001**
*p* -Value for palatal cervical margin	**<0.0001**	**<0.0001**	0.0340

Note: Bold *p* -Values indicate statistical significance.

## Discussion

Laminate veneers are the conservative treatment option for unaesthetic anterior teeth. Continued development of dental ceramics and composites offers clinicians many viable treatment options for creating highly aesthetic and functional veneers. 3 The evolution of dental materials, particularly ceramics and adhesive systems, has significantly enhanced aesthetic outcomes and contributed to improved patient satisfaction and self-esteem. Clinicians must remain updated on the latest developments in aesthetic materials, including their physical properties, indications, and handling techniques, to ensure predictable and successful clinical outcomes. 17 18

The most important criterion dentists use to evaluate the clinical acceptability of cast restorations is their marginal fit. Marginal discrepancies have been proven responsible for plaque accumulation, which is the primary causative factor in the etiology of periodontal disease and caries, the main cause of frequently encountered aesthetic problems. 19 20 In this study a perpendicular measurement at the margin from casting to the finish line was considered as vertical marginal discrepancy.

Extracted human maxillary central incisor was used in the present study, as it is the most common tooth indicated for veneers. Cervically, chamfer finish line was prepared as it is a conservative margin, provides increased bulk of porcelain giving adequate strength, avoids over-contouring, and provides good marginal seal, in accordance with other studies. 3 5 6 7 8 9 10 11 12 13 14 15 16 17 18 19 20 21 22

The incisal overlap design used in this study reflects a clinically relevant and widely adopted technique in anterior veneer preparation. It allows enhanced aesthetic control of incisal translucency and contributes to improved resistance to incisal chipping due to better load distribution. However, it is important to acknowledge that there is ongoing debate in the literature regarding the influence of preparation design on marginal adaptation. Although certain *in vitro* studies have shown that butt–joint margins may result in superior marginal fit due to reduced geometric complexity, 14 22 other reports support overlap designs for their functional and aesthetic advantages, especially when combined with modern adhesive protocols. 5 23 Future studies comparing marginal fit across different preparation geometries using identical materials and methods would help clarify these differences.

Two methods of evaluating the marginal discrepancy are measuring the sectioned specimen and measurement made by direct visualization under stereomicroscope. The former method is more destructive whereas the second method is non-destructive; also, it provides several measuring points. In the current study, stereomicroscope was used, which was in agreement with the other studies. 24 25 26 The mounted prepared tooth in acrylic block was stabilized in jig assembly and six points were evaluated directly under stereomicroscope using image analysis software, which is in accordance with the study of Baig et al. 27

The highest mean discrepancy of vertical marginal fit at cervical margins was observed in ICV followed by CAD-CAM veneers and PCV. Similar results were observed for palatal margins as well. When compared with palatal margin, cervical margins showed greater marginal discrepancies in all the groups. These results were in agreement with the study done by Hannig et al. 28 Poor marginal fit of the composite veneers as compared with other groups could be attributed to the polymerization shrinkage and viscosity of the composite material. 29

The results showed that PCV showed overall least marginal discrepancy followed by CAD-CAM veneers and more was seen in ICV. There was a statistically significant difference among the three groups ( *p* < 0.0001), which was in accordance with other studies. 30 31 32 This may be due to the difference in the fabrication procedures as well as the materials used and the fabrication method of PCV using lost wax technique for the processing.

It is important to note that the marginal fit in this study was assessed prior to cementation. Although this approach allowed a standardized comparison of veneer fit based solely on fabrication techniques and materials, it does not replicate the actual clinical scenario. In practice, the use of resin-based luting agents may alter marginal adaptation due to variables such as film thickness, flow characteristics, and polymerization shrinkage. Previous studies have shown that marginal discrepancies can increase following cementation, especially in areas of resistance or where excess cement cannot escape freely. Therefore, although our findings provide insight into the intrinsic adaptation of the veneers, clinical outcomes may vary once cement is applied.

In the present study, the marginal discrepancy values observed in the pressable ceramic and CAD/CAM groups were within the clinically acceptable threshold of ≤120 µm, which aligns with findings from recent investigations. 10 33 However, the ICV group exhibited values that exceeded this limit, suggesting a higher risk for long-term marginal failure. This reinforces the importance of both material selection and fabrication method in achieving optimal marginal adaptation.

On the other hand, CAD-CAM milled veneers resulted in greater marginal discrepancy than expected. Similar results were found in the study done by Reich et al. 34 This may be due to the software limitations in designing the restorations, hardware limitations of camera and scanning equipment, and errors produced by milling machines in the technique that resulted in greater marginal discrepancy. The results of the present study are in contrast to those obtained in a study by Yüksel and Zaimoğlu, in which they found lower marginal discrepancy values in CAD/CAM-fabricated ZrO _2_ copings as compared with heat-pressed lithium-disilicate copings. 35

The cervical marginal fit values of PCV and ICV were found to be significantly better, which was in accordance with the study performed by Harasani et al. 36

In our study, the mean values of marginal discrepancy for PCV and CAD-CAM veneers were within the clinically acceptable range, whereas ICV (134.12 μm) was not clinically acceptable. The marginal openings should be in the range of 7 to ≤120 μm to be acceptable clinically. 9 37

It must be noted that the marginal fit values in this study were assessed without final cementation. Although non-cemented evaluation allows for isolation of fabrication-related discrepancies, it does not account for the influence of the cement layer, which may alter marginal adaptation post-cementation. Prior studies have shown that cementation can increase marginal discrepancies due to the viscosity, flow, and film thickness of the luting agent.

This study has several limitations that should be acknowledged. First, the evaluation of marginal adaptation was conducted under *in vitro* conditions, which may not fully replicate the clinical environment. The surface characteristics of an extracted tooth differ from those of a vital tooth in terms of hardness, wettability, and structural integrity, potentially influencing the adaptation outcomes. Additionally, the veneers were assessed without cementation, which limits the evaluation of real-time clinical marginal fit. The thickness, viscosity, and flow behavior of the luting agent can significantly impact marginal integrity, especially under the dynamic forces involved during clinical setting. Therefore, future *in vivo* studies incorporating cementation protocols are recommended to validate these findings.

One of the limitations is the use of a single human maxillary central incisor for all specimens. Although this approach ensured strict standardization of the preparation and eliminated anatomical variability, it may reduce the generalizability of the results to other tooth types and clinical scenarios. Nonetheless, this methodological choice was made to enhance internal validity and ensure consistency across experimental groups. Another limitation of the present study is the restricted number of measurement points used to assess marginal fit. Although three points on each of the labial and palatal surfaces were analyzed to maintain methodological consistency, this does not represent the full circumferential margin. Variability in marginal adaptation at interproximal or curved surfaces may have been overlooked. Future investigations should include additional measurement sites or utilize digital scanning for a more detailed evaluation. Additionally, the use of a single operator for all measurements precluded the evaluation of inter-examiner reliability. Although this ensured consistency, it may also introduce operator-dependent bias. The stereomicroscope was calibrated prior to use; however, future studies should consider including multiple calibrated observers and calculating inter-rater agreement to further enhance reliability.

## Conclusion

Within the limitation of present study, PCV showed the best marginal fit at both cervical and palatal margins followed by CAD/CAM ceramic veneers, whereas ICV showed poorest marginal fit. The marginal discrepancy values were within the clinically acceptable range for PCV and CAD/CAM ceramic veneers. So, it is of paramount importance that the dentist should chose the aesthetic veneer materials wisely taking into account their physical features, especially the marginal fit.
